# Anti-Müllerian hormone as a marker of steroid and gonadotropin action in the testis of children and adolescents with disorders of the gonadal axis

**DOI:** 10.1186/s13633-016-0038-2

**Published:** 2016-10-28

**Authors:** Nadia Y. Edelsztein, Romina P. Grinspon, Helena F. Schteingart, Rodolfo A. Rey

**Affiliations:** 1 Centro de Investigaciones Endocrinológicas “Dr. César Bergadá” (CEDIE), CONICET – FEI – División de Endocrinología, Hospital de Niños Ricardo Gutiérrez, Buenos Aires, Argentina; 2 Departamento de Ecología, Genética y Evolución, Facultad de Ciencias Exactas y Naturales, Universidad de Buenos Aires, Buenos Aires, Argentina; 3 Departamento de Biología Celular, Histología, Embriología y Genética, Facultad de Medicina, Universidad de Buenos Aires, Buenos Aires, Argentina

**Keywords:** Testis, Sertoli, Cryptorchidism, Puberty, Disorders of sex development

## Abstract

In pediatric patients, basal testosterone and gonadotropin levels may be uninformative in the assessment of testicular function. Measurement of serum anti-Müllerian hormone (AMH) has become increasingly widespread since it provides information about the activity of the male gonad without the need for dynamic tests, and also reflects the action of FSH and androgens within the testis. AMH is secreted in high amounts by Sertoli cells from fetal life until the onset of puberty. Basal AMH expression is not dependent on gonadotropins or sex steroids; however, FSH further increases and testosterone inhibits AMH production. During puberty, testosterone induces Sertoli cell maturation, and prevails over FSH on AMH regulation. Therefore, AMH production decreases. Serum AMH is undetectable in patients with congenital or acquired anorchidism, or with complete gonadal dysgenesis. Low circulating levels of AMH may reflect primary testicular dysfunction, e.g. in certain patients with cryptorchidism, monorchidism, partial gonadal dysgenesis, or central hypogonadism. AMH is low in boys with precocious puberty, but it increases to prepubertal levels after successful treatment. Conversely, serum AMH remains at high, prepubertal levels in boys with constitutional delay of puberty. Serum AMH measurements are useful, together with testosterone determination, in the diagnosis of patients with ambiguous genitalia: both are low in patients with gonadal dysgenesis, including ovotesticular disorders of sex development, testosterone is low but AMH is in the normal male range or higher in patients with disorders of androgen synthesis, and both hormones are normal or high in patients with androgen insensitivity. Finally, elevation of serum AMH above normal male prepubertal levels may be indicative of rare cases of sex-cord stromal tumors or Sertoli cell-limited disturbance in the McCune Albright syndrome.

## Background

In the adult male, the appraisal of the endocrine function of the gonadal axis usually relies on the assessment of serum levels of gonadotropins, testosterone and inhibin B. In pediatric ages, basal testosterone and gonadotropin levels may be largely uninformative. In fact, gonadotropin and testosterone secretion is active only during 3 to 6 months after birth in the male; thereafter, their serum levels remain very low or undetectable until the onset of puberty [1]. However, the use of non-classical biomarkers, like anti-Müllerian hormone (AMH), has become increasingly widespread since it not only informs about the activity of the male gonad without the need for dynamic tests but also reflects the action of FSH and androgens within the gonad [2]. This review will address the usefulness of AMH as a biomarker of testicular function in prepubertal and adolescent males, based on the knowledge of the endocrine regulation of testicular AMH secretion during pre- and post-natal development.

## Developmental physiology of the hypothalamic-pituitary-testicular axis

Testicular function is mainly regulated by the pituitary gonadotropins LH and FSH, which in turn depend on gonadotropin-releasing hormone (GnRH) action, from the hypothalamus. This hypothalamic-pituitary-gonadal axis evolves throughout development, from fetal life through adulthood. Specific maturational changes take place both in these organs as a whole and in the different cell types that make them up.

While sperm production has classically been the focus of adult reproductive function, somatic cells are crucial for the maintenance of spermatogenesis and gamete production. In the interstitial tissue, Leydig cells synthesize androgens and the insulin-like factor 3 (INSL3) [3], whereas in the seminiferous tubules, Sertoli cells regulate the nutrients and factors that reach the germ cells by means of the blood-testis barrier. Sertoli cells not only regulate the inflow of external substances, but also produce several substances which are critical to the proper progression of spermatogenesis [4]. Therefore, it appears evident that the assessment of gonadal function and the definition of male hypogonadism should rely on the understanding of normal testicular physiology resulting from the integrated function of the tubular and interstitial compartments, and its developmental changes from fetal life through maturity [5].

## Sertoli cells as the most active population in the developing testis

Unlike the adult testis, where germ cells represent most of the gonadal size and Leydig cells are the most active endocrine cell population, in the prepubertal testis, Sertoli cells are the most numerous [6] and active testicular cell population [7, 8]. Even though Sertoli cells remain active during infancy and childhood, the testes have been erroneously considered as quiescent due to the reduced activity of the hypothalamic-gonadotrope axis. This activity is clearly reflected on the high levels of serum AMH and inhibin B.

**Fig. 1:**
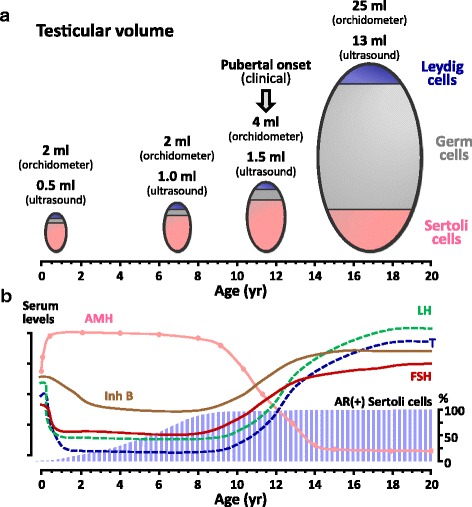
Developmental physiology of the testis in postnatal life. **a**: Testicular volume increases slightly during infancy and childhood (from birth to the age of 8–10 yr), as measured by ultrasonography, mainly due to the increase of the Sertoli cell population. After pubertal onset, clinically defined by a testicular volume of 4 ml as measured by comparison with the orchidometer, testicular volume increases drastically due to the onset of pubertal spermatogenesis, which requires androgen-dependent Sertoli cell maturation. **b**: Schematic serum levels of gonadotropins (FSH and LH), testosterone (T), inhibin B (Inh B) and AMH from birth through adulthood (left axis) and percentage of Sertoli cells expressing the androgen receptor (AR, right axis). *Reprinted, with permission, from Rey et al.* [85]*, copyright 2009 Wiley-Liss, Inc.*

Earlier in development, during fetal life and early infancy, the active hypothalamic-gonadotrope axis has effects on the seminiferous cords, reflected in the proliferation of both immature germ and Sertoli cells [9]. Sertoli cell proliferation, essentially dependent on FSH, results in a moderate increase in testicular volume, which cannot be detected by palpation [6, 10–12] but is clearly measurable by ultrasonography [13] (Fig. 1a).

It is around the onset of puberty that Sertoli cells undergo major morphological and physiological changes, leading to the switch from a proliferative, immature state, to a quiescent, mature one. Morphologically, there are changes in the nucleus and nucleolus [14]. The blood-testis barrier becomes distinct, creating two separate compartments within the tubules. Germ cells in the adluminal compartment become dependent on the function of the, now mature, Sertoli cells [15]. These maturational changes observed in Sertoli cells are induced essentially by an increase of intratesticular testosterone concentration early in pubertal development [14, 16]. Interestingly, Sertoli cells do not show maturational changes, in spite of the active androgen testicular production, during fetal and neonatal periods of life. This is due to the fact that before the age of 1 yr in humans [17, 18], the androgen receptor is not expressed in Sertoli cells (Fig. 1b), as experimentally confirmed in mice [19, 20].

## AMH as a marker of prepubertal Sertoli cells: physiological concepts

AMH, also known as Müllerian Inhibiting Substance (MIS), is a glycoprotein dimer belonging to the transforming growth factor β (TGF-β) family [21, 22], which plays a major role in fetal sex differentiation by inducing the regression of the Müllerian ducts.

In the male, AMH expression begins when the seminiferous cords differentiate in the fetus [23], and remains high until puberty [23–26] (Fig. 1b and Table 1). The onset of AMH expression in fetal life is independent from gonadotropins, and involves several transcription factors. Initially, SOX9 binds to the AMH promoter [27, 28] and triggers its expression; subsequently, other transcription factors, such as SF1 [27, 29, 30], GATA4 [30, 31] and WT1 [32], further increase AMH production.

**Table 1:** Serum AMH levels in normal boys

Age	Serum AMH	
	pmol/l^a^	ng/ml^a^
<14 days	250–1000	35–140
15 days – 6 months	400–1500	55–210
6 months – 2 years	600–2300	85–320
2–9 years	400–1800	55–250
9–18 years:		
Tanner 1	250–1400	35–200
Tanner 2	70–1000	10–140
Tanner 3	30–400	4–55
Tanner 4	30–160	4–22
Tanner 5	30–150	4–21
Adults	25–130	3–18

^a^Reference levels are taken from refs. [24, 25, 26]. For calculations, 1 ng/ml is equivalent to 7.14 pmol/l

Because AMH is exclusively secreted into the circulation by Sertoli cells [33, 34], it has become one of the most useful markers to study testicular function during the prepubertal period in the male [35–37]. In the female, AMH is produced by ovarian granulosa cells of primary and small growing follicles up until transition to menopause [38–41].

### AMH as a marker of FSH action in the testis

**Fig. 2:**
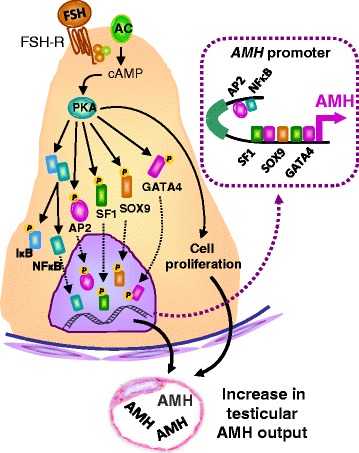
AMH as a marker of FSH action in the prepubertal testis. FSH provokes Sertoli cell proliferation and increases AMH transcription in each Sertoli cell through the classical FSH receptor (FSH-R) transduction pathway involving Gsα protein, adenylyl cyclase (AC) and stimulation of protein kinase A (PKA) activity, leading to an increased AMH promoter activity induced by transcription factors SOX9, SF1, GATA4, NFκB and AP2

Once AMH expression is triggered independently of gonadotropins in fetal and postnatal life, FSH further increases testicular AMH output by inducing Sertoli cell proliferation and up-regulating AMH transcription (Fig. 2), which explains why patients with congenital central (hypogonadotropic) hypogonadism have low AMH serum levels that increase after treatment with exogenous FSH [42, 43]. These results clearly demonstrate that serum AMH is an adequate marker of FSH action in the prepubertal testis. The usefulness of serum AMH levels as an indicator of FSH action has also been studied in rodents: the absence of FSH stimulation during fetal and neonatal life results in low levels of AMH due to a decrease in Sertoli cell number and AMH expression, correlating also with smaller testes [44]. FSH administration to neonatal mice provokes an increase in testicular volume and in AMH transcription through the classical FSH receptor transduction pathway involving Gsα protein, adenylyl cyclase and stimulation of protein kinase A (PKA) activity, leading to the involvement of the aforementioned transcription factors SOX9, SF1, GATA4, and also of NFκB and AP2 [2, 20, 44, 45] (Fig. 2).

### AMH as a marker of androgen action in the testis

**Fig. 3:**
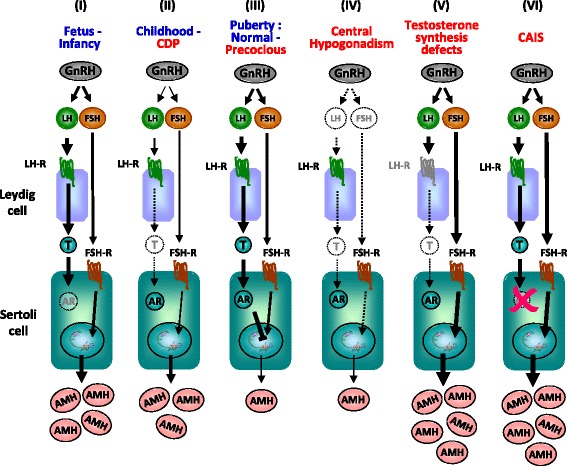
Regulation of testicular AMH production by FSH and testosterone in normal and pathological conditions. Basal AMH production is independent of gonadotropins or androgens; however, FSH stimulates and testosterone inhibits AMH expression. In the ***fetal period and during the first months of postnatal life (I)***, the hypothalamic-gonadotrope is active: FSH stimulates AMH production, whereas testosterone cannot inhibit it because Sertoli cells do not yet express the androgen receptor. During ***childhood***, and in boys >14 years-old with ***constitutional delay of puberty (II)***, the hypothalamic-gonadotrope is “quiescent”, resulting in little or no effect on basal AMH production. In boys with ***normal or precocious puberty (III)*** with high intratesticular androgen concentrations (central precocious puberty, testotoxicosis, Leydig cell tumors), testosterone inhibition overrides FSH stimulation, resulting in a decrease in serum AMH. In patients with ***central hypogonadism (IV),*** only basal AMH production is observed, with no further stimulation or inhibition. In patients with disorders of sex development due to ***androgen synthesis defects (V)*** or ***androgen insensitivity (VI)***, the positive effect of FSH cannot be antagonized by testosterone, resulting in high AMH production in infancy and pubertal age. AR: androgen receptor; CAIS: complete androgen insensitivity syndrome; CDP: constitutional delay of puberty; FSH-R: FSH receptor; LH-R: LH receptor; T: testosterone

At the onset of puberty, AMH serum levels start declining, as compared to prepubertal levels, and continue to decrease throughout puberty [46] (Fig. 1b and Table 1), as a consequence of the negative effect exerted by intratesticular testosterone via the androgen receptor [20, 47] (Fig. 3). The androgen-mediated downregulation of AMH expression occurs concomitantly with the appearance of meiotic germ cells in the seminiferous tubules, indicating Sertoli cell maturation [20, 47, 48]. The inhibitory effect of androgens on AMH expression overrides the FSH-dependent stimulation in normal puberty (Fig. 3). The androgen-dependent inhibition of AMH has also been observed in central precocious puberty and in male-limited gonadotropin-independent precocious puberty (testotoxicosis), clearly indicating that androgens are responsible for AMH down-regulation independently of gonadotropin levels [46]. Interestingly, the decline of AMH levels reflects an increase in intratesticular, and not necessarily circulating, testosterone concentration, as observed in the earliest stages of puberty [26, 46]. Conversely, in patients with central hypogonadism treated with exogenous testosterone, serum AMH remains high indicating that intratesticular androgen concentration is low [49]. This is in line with the lack of increase in testicular volume, since pubertal and adult spermatogenesis needs sufficient intratesticular androgen concentration to develop. Similarly, in cases of constitutional delay of puberty [50, 51] or of defective androgen production or sensitivity [20, 52, 53], the lack of androgen production or action results in the maintenance of high AMH levels (Fig. 3).

Androgen-mediated AMH down-regulation is also not observed in fetal life and during the first year of postnatal life (Fig. 3), even in patients with precocious puberty, owing to the above-mentioned physiological androgen insensitivity of Sertoli cells, which is consequence of the lack of androgen receptor expression in Sertoli cells in those periods of life [17, 18, 54, 55].

Ever since Alfred Jost postulated the existence of AMH [56], it has been referred to as the fetal testicular hormone guiding the regression of the Müllerian ducts in the male fetus. The biological reasons for ongoing expression of AMH throughout childhood have been the source of many debates. Nonetheless, AMH detection in serum has become a very powerful tool in pediatrics. In the following part of this review, we aim to summarize the main conditions in which AMH can be used as a proper marker of Sertoli cell function in boys.

## Serum AMH in the diagnosis of conditions affecting testicular function

### Cryptorchidism

Cryptorchidism is a clinical sign with many different etiologies [57, 58]. It may be a consequence of primary (usually called hypergonadotropic) or central (hypogonadotropic) hypogonadism, or even result from anatomical defects of the inguinal region or the abdominal wall (i.e. not due to hypogonadism). Cryptorchidism may be associated with normal or impaired Sertoli cell function [59, 60] (Table 2). In boys with bilateral cryptorchidism, AMH is low in approximately 75 % of those with non-palpable gonads and 35 % of those with inguinal gonads, indicating Sertoli cell dysfunction [61].

**Table 2:** Serum AMH levels according to clinical presentation

	Serum AMH			
Clinical sign	Undetectable	Low	Normal	High
Cryptorchidism	Anorchidism (Testicular regression, bilateral gonadectomy) PMDS - *AMH* mutation	Primary hypogonadism (testicular dysgenesis syndrome) Central hypogonadism	Rules out testicular dysgenesis PMDS - *AMHR* mutation	--
Micropenis	Fetal testicular regression	Primary hypogonadism Central hypogonadism	Malformative micropenis	--
Absence of puberty	Testicular regression Bilateral gonadectomy	Primary hypogonadism Central hypogonadism	Constitutional delay of puberty	--
Precocious pubertal signs	--	Central Precocious Puberty Testotoxicosis Leydig cell tumor	Congenital adrenal hyperplasia Adrenal androgen-secreting tumors Exogenous androgen exposure	--
Prepubertal macro-orchidism	--	--	--	McCune-Albright syndrome Sex-cord stromal tumors
DSD	46,XY Complete gonadal dysgenesis	46,XY Partial gonadal dysgenesis Sex-chromosome gonadal dysgenesis Ovotesticular DSD	Androgen synthesis defects Androgen insensitivity 46,XY Malformative DSD 46,XX male (Testicular DSD)	Androgen synthesis defects Androgen insensitivity

Serum AMH levels are considered low, normal or high as compared to those expected for age in normal boys

*AMH-R* AMH receptor, *DSD* disorders of sex development, *PMDS* persistent Müllerian duct syndrome

### Non-palpable gonads

In patients with non-palpable gonads, it is necessary to determine whether there is intraabdominal functional testicular tissue. The utility of gonadotropins, as indirect markers, is limited since they may be normal even in anorchid children [1]. Conversely, in boys with non-palpable gonads detectable serum AMH levels are highly predictive of the existence of testicular tissue while an undetectable AMH value is indicative of anorchidism [33, 34] (Table 2). An extremely rare exception is the Persistent Müllerian Duct Syndrome caused by *AMH* gene mutations, which may explain the finding of undetectable serum AMH in a boy with abdominal testes [62]. Vanishing or regression of testicular tissue occurring in the second half of fetal life does not preclude virilization, but micropenis and hypoplastic scrotum occur (Table 2). Serum AMH is low or undetectable, according to the amount of remaining functional testicular tissue [33, 34].

### Monorchidism

Monorchidism is the presence of a solitary testis, which may undergo a compensatory volume increase. There is a dissociated capacity of the remaining testis to compensate for the absence of the other gonad: while Leydig cell function is largely compensated, lower AMH and higher FSH in monorchid boys indicate that Sertoli cell proliferation and function is insufficient to fully compensate the function of the absent one [63].

### Klinefelter syndrome

No overt signs of hypogonadism are evident before puberty in Klinefelter syndrome, a sex-chromosome aneuploidy with late-onset testicular dysgenesis. Serum AMH is normal during childhood and early puberty, in correlation with normal inhibin B and FSH, indicating that Sertoli cell function is preserved until advanced stages of puberty [64, 65]. At the onset of puberty, like in normal boys, androgens provoke a physiological decrease in serum AMH also in patients with Klinefelter syndrome. However, in the latter, Sertoli cell function deteriorates progressively from mid-puberty, resulting in extremely low or undetectable AMH, in coincidence with undetectable inhibin B, very high FSH levels and small testis volume. Germ cell degeneration has been described already in early fetal development with a clear progression during postnatal life, mainly after pubertal onset [66].

### Cryptorchidism and micropenis: suspicion of central hypogonadism

During the neonatal period and infancy, some clinical features associated with cryptorchidism, like micropenis and microorchidism, or the coexistence of anosmia or other pituitary hormone deficiencies are suggestive of central hypogonadism. Serum AMH is below the normal range in most cases of isolated central hypogonadism and of multiple pituitary hormone deficiency [42, 43, 49] (Table 2 and Fig. 3), although normal AMH levels do not rule out the diagnosis [67]. The lack of FSH stimulation during fetal and neonatal life is responsible for the decreased Sertoli cell numbers and low AMH expression in patients with congenital hypogonadotropic hypogonadism.[44, 45] The increase in serum AMH in those patients receiving FSH may be useful to monitor treatment efficacy [42, 43, 49].

### Pubertal delay

Sertoli cells markers have been assessed to distinguish between constitutional delay of puberty and central hypogonadism. AMH is within normal prepubertal levels in boys with constitutional delay of puberty, reflecting a eugonadal state in these patients [51]. In untreated patients of pubertal age with congenital central hypogonadism, serum AMH levels are above those expected for age –reflecting that intratesticular testosterone is too low to inhibit AMH– but below those expected for Tanner stage 1, indicating that Sertoli cells have not been exposed to FSH [49, 68] (Table 2 and Fig. 3). Treatment with recombinant FSH provokes an increase in serum AMH, whereas further administration of hCG results in an elevation of intratesticular androgen levels and a decline in AMH [42, 49]. Conversely, down-regulation of AMH is less notorious when patients receive exogenous testosterone, probably due to the lower intratesticular androgen levels obtained with this treatment [49].

### Precocious puberty

**Fig. 4:**
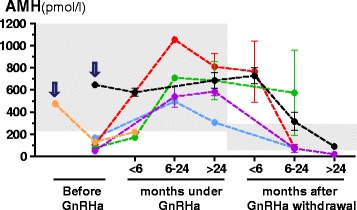
Serum AMH as a marker of increased intratesticular androgen activity in patients with precocious puberty. AMH levels at diagnosis, during and after treatment in six patients with central precocious puberty. Each color line represents a different patient. Serum AMH is low for age in four of the boys with precocious puberty, indicating that there is a high intratesticular testosterone concentration that inhibits AMH expression. When testosterone production is curtailed by treatment with a GnRH analogue, serum AMH recovers prepubertal levels until treatment is withdrawn. In the remaining two cases (*arrows*), diagnosed before the age of 1 year, the explanation for normal prepubertal AMH levels at diagnosis, indicating a lack of AMH expression in spite of high androgen levels, is that Sertoli cells do not yet express the androgen receptor at that age. The shaded areas represent normal reference AMH levels for age. *Reprinted, with modifications, from: Copyright 2013 R.P. Grinspon et al* [18]

Like in normal puberty, serum AMH declines in boys with central or gonadotropin-independent precocious puberty, showing the well-known inhibition exerted by androgens on Sertoli cell AMH production (Table 2 and Figs. 3 and 4). Low AMH together with increased testosterone levels for chronological age are suggestive of precocious testicular maturation.

Serum AMH determination may be particularly helpful in the diagnostic workup of boys with incipient signs of precocious puberty, e.g. testis volume increase from 2 to 3 ml with or without penile enlargement, in whom basal gonadotropin and testosterone levels are not yet informative. As already mentioned, the decline in serum AMH is an early biochemical sign of the increase in intratesticular testosterone concentration [69]. In infants below the age of 1 yr, AMH may not be useful (Figs. 3 and 4): serum levels are normal, owing to the lack of androgen receptor expression in Sertoli cells at that age, which makes this particular cell population of the testis transiently insensitive to androgens [18].

AMH may also be useful to monitor effectiveness during treatment with GnRH analogues, ketoconazole or anti-androgens. The decrease in testosterone production or action is reflected in the recovery of prepubertal AMH levels [18, 46]. Interestingly, lack of adherence to treatment resulting in intermittent inhibition of testosterone production can be suspected when AMH does not normalize [46].

### Prepubertal macro-orchidism

Although precocious puberty is one of the classic components of McCune-Albright syndrome, macro-orchidism in the absence of androgen-dependent secondary sexual characteristics has also been reported in boys [70, 71]. In some cases, increased Sertoli cell proliferation was detected by the presence of small hyperechogenic foci in ultrasound imaging [72]. The typical somatic activating mutation in the *GNAS1* gene, encoding for the Gsα protein involved in the FSH receptor transduction pathway, was present only in Sertoli cells, thus resulting in isolated Sertoli cell hyperfunction with Sertoli cell hyperplasia and increased AMH (Table 2), without activating Leydig cells [70].

### Sex-cord stromal tumors

AMH immunohistochemistry is useful to identify the sex-cord stromal origin in testicular tumors. AMH expression has been shown in overt Sertoli cell tumors [73, 74], in large cell calcifying Sertoli cell tumors frequently associated with Peutz-Jeghers syndrome [74], in primary or metastatic granulosa cell tumors of the testis [73], and in intratubular Sertoli cell proliferations, which has been suggested to represent an “in situ” or early stage of Sertoli cell tumors [74]. Although a single determination of serum AMH may not be helpful to establish the initial diagnosis of the tumors in most of pediatric cases, because high AMH is normally found in the prepubertal boy, increasing AMH levels may be suggestive of a progressive lesion.

### Cancer survivors

Chemotherapy and radiotherapy affect primarily germ cells of the testis, while steroid secreting Leydig cells are less affected. Sertoli cell function has not been extensively studied in cancer survivors. Two reports including few patients treated with poly-chemotherapy or hematopoietic cell transplantation for medulloblastoma or posterior fossa ependymoma have shown AMH below normal range for age [75, 76], whereas our group could not demonstrate any decrease in serum AMH in a large series of patients with Acute Lymphoblastic Leukemia or Lymphoblastic Lymphoma who received poly-chemotherapy [77].

### Ambiguous genitalia

When a 46,XY newborn is born with ambiguous or female genitalia, *i.e.* a 46,XY disorder of sex development (DSD), causes of insufficient virilization should be investigated [78]. 46,XY DSD may result from disorders affecting both tubular and interstitial testicular compartments, like gonadal dysgenesis, or from a condition affecting only the interstitial compartment, like Leydig cell aplasia or steroidogenic enzyme defects. While testosterone is low in both situations, serum AMH is helpful to establish a differential diagnosis since it is low in patients with gonadal dysgenesis but normal or high in patients with isolated androgen deficiency [53, 62, 78–80] (Table 2 and Fig. 3).

Alternatively, the action of androgen in target tissue may be affected in the androgen insensitivity syndrome. In these patients, both Sertoli and Leydig cell activity is preserved, as reflected by normal to elevated serum AMH and androgen levels [53, 62, 78–80] (Table 2 and Fig. 3).

In boys with isolated hypospadias, AMH and testosterone are usually normal, indicating that there is no testicular dysfunction, and a malformative DSD should be suspected. When hypospadias is associated with other clinical manifestations of undervirilization like cryptorchidism, a higher risk of abnormal hormone secretion by the gonads or androgen end-organ defects exists [81, 82].

The Persistent Müllerian Duct Syndrome is a rare form of 46,XY DSD usually diagnosed by the unexpected finding of Müllerian duct remnants during a surgical procedure for cryptorchidism. Serum AMH levels are useful to differentiate its etiology, with normal serum AMH in patients with AMH receptor mutations and extremely low or undetectable AMH levels in patients with AMH gene mutations [62] (Table 2).

In 46,XX DSD patients with ambiguous external genitalia, AMH levels above the normal female range exclude the diagnosis of congenital adrenal hyperplasia, aromatase defects or virilizing tumors, and are highly suggestive of an Ovotesticular DSD [53, 78, 83].

In fully virilized 46,XX DSD patients (XX males), AMH and testosterone are in the normal male range (Table 2), indicating that Leydig and Sertoli cells are not primarily affected [53, 78]. However, germ cells fail to progress through meiosis and undergo apoptosis at puberty, associated with low testicular volume [84].

## Conclusions

Serum AMH is an extremely helpful marker for assessing testicular function in pediatric patients. In 46,XY patients with non-palpable gonads and in newborns with DSD, serum AMH is informative about the existence and functional capacity of testicular tissue. Serum AMH levels are commensurate with the amount of functional Sertoli cells present in prepubertal patients, including those with micro- or macro-orchidism, or ovotesticular DSD. Serum AMH is also a reliable marker of FSH action in the prepubertal testis, both in basal conditions to diagnose central hypogonadism and to monitor FSH treatment. Finally, declining serum AMH is indicative of effective androgen action within the seminiferous tubules, and therefore a useful marker in the diagnosis and follow-up of patients with precocious or delayed puberty.

## Abbreviations

AMH: Anti-Müllerian hormone
AMH-R: AMH receptor
AR: Androgen receptor
CAIS: Complete androgen insensitivity syndrome
CDP: Constitutional delay of puberty
DSD: Disorders of sex development
FSH-R: FSH receptor
GnRHa: GnRH analogue
INSL3: Insulin-like factor 3
LH-R: LH receptor
MIS: Müllerian inhibiting substance
PKA: Protein kinase A
PMDS: Persistent Müllerian duct syndrome
T: Testosterone
TGFβ: Transforming growth factor β
